# Evidence against egocentric prediction during language comprehension

**DOI:** 10.1098/rsos.231252

**Published:** 2023-12-13

**Authors:** Ruth E. Corps, Fang Yang, Martin J. Pickering

**Affiliations:** ^1^ Psychology of Language Department, Max Planck Institute for Psycholinguistics, Nijmegen, The Netherlands; ^2^ Department of Psychology, University of Edinburgh, Edinburgh, UK

**Keywords:** prediction, perspective-taking, visual-world paradigm, gender identity

## Abstract

Although previous research has demonstrated that language comprehension can be egocentric, there is little evidence for egocentricity during prediction. In particular, comprehenders do not appear to predict egocentrically when the context makes it clear what the speaker is likely to refer to. But do comprehenders predict egocentrically when the context does not make it clear? We tested this hypothesis using a visual-world eye-tracking paradigm, in which participants heard sentences containing the gender-neutral pronoun *They* (e.g. *They would like to wear…*) while viewing four objects (e.g. tie, dress, drill, hairdryer). Two of these objects were plausible targets of the verb (tie and dress), and one was stereotypically compatible with the participant's gender (tie if the participant was male; dress if the participant was female). Participants rapidly fixated targets more than distractors, but there was no evidence that participants ever predicted egocentrically, fixating objects stereotypically compatible with their own gender. These findings suggest that participants do not fall back on their own egocentric perspective when predicting, even when they know that context does not make it clear what the speaker is likely to refer to.

## 1. Introduction

There is much research demonstrating that language comprehension can be egocentric, with listeners initially comprehending from their own perspective (see [1] for a review). But there is little evidence for egocentricity during prediction [2,3]. However, these studies have used a visual-world paradigm in which the comprehender sees only one entity that the speaker is likely to refer to, and other entities that are implausible. As a result, the comprehender has a strong sense of what type of entity the speaker is likely to refer to. In this paper, we ask what happens when the comprehender does not know what the speaker is likely to refer to because there is more than one plausible entity. Does the comprehender now predict from their own (egocentric) perspective?

There is much evidence that speakers predict what a speaker is likely to say. For example, Altmann & Kamide [4] found that participants fixated a picture of a cake (rather than other inedible objects) earlier and for longer when they heard a speaker say *The boy will eat…* compared to when they heard the speaker say *The boy will move*. These findings suggest that comprehenders can predict the meaning of upcoming words—they used the semantics of the verb to predict which object was most likely to be mentioned next (i.e. edible objects).

Other studies suggest that comprehenders can also incorporate world knowledge (such as another person's perspective) into their predictions. In one study, Heller *et al.* [5] presented participants with displays containing two pairs of size-contrasting objects. One pair (e.g. a big bowl and a small bowl) was visible to both participants (and the comprehender knew the speaker could see them). One object from the other pair was also visible to both participants (a big car), while the other object (a small car) was visible only to the comprehender. On hearing *The big…* , comprehenders fixated the big bowl more than the big car, suggesting they considered the speaker's perspective.

Creel [6] found that participants who were explicitly taught a character's colour preferences (e.g. a female speaker preferred pink) predictively fixated objects that matched the speaker's colour preference. Furthermore, Borovsky & Creel [7] familiarized participants with two speakers (e.g. a pirate and a princess) whose roles were strongly associated with particular objects. Participants predictively fixated objects compatible with the speaker's role; for example, if they heard the pirate speaking then they fixated the sword and the ship more than the wand and the carriage. Importantly, perspective was salient in these studies—in Creel [6] and Borovsky & Creel [7], participants could be confident which objects the speaker would refer to when they heard the first word of the sentence.

Corps *et al*. [2] relied on gender stereotypes, rather than explicitly familiarizing participants with particular perspectives, to investigate the role of perspective in prediction. Participants listened to male and female speakers produce sentences such as *I would like to wear the nice…* while viewing four objects. Two of these objects were semantic associates of the verb (targets; e.g. a tie and a dress), while two were not associates (distractors; e.g. a drill and a hairdryer). One target (the dress) and one distractor (the hairdryer) were stereotypically feminine (as rated by a separate group of participants), while the other target (the tie) and distractor (the drill) were stereotypically masculine. Participants looked at associates of the verb (the tie and the dress) more than objects that were not associates (the drill and the hairdryer) within 519 ms after verb onset, suggesting participants predicted associatively. They also predicted consistently—that is, consistent (or in accord) with their beliefs about what the speaker would actually say based on the speaker's gender identity. But they did so later: participants fixated associates stereotypically compatible with the speaker's gender (the tie for a male speaker; the dress for a female speaker) more than associates that were not, from 641 ms after verb onset. The second and third experiments provided further evidence that comprehenders integrate perspective into their predictions, using sentences in which the word *I* was replaced with *You* in Experiment 2 or with a name that was stereotypically masculine (*James*) or feminine (*Kate*) in Experiment 3. Thus, the comprehender predicted from the perspective of the agent of the sentence (note the agent and the speaker are the same in Experiment 1).

But the agent's perspective was apparent in Corps *et al*. [2]—it was cued using the speaker's voice, the sentence pronoun, or a character's name. Even though it was less salient in Corps *et al*. than in Creel [6] and Borovsky & Creel [7], there was only one on-screen entity that was compatible with the linguistic context and with gender stereotypes (e.g. a tie if a male speaker said *I would like to wear*…). But what happens when there is more than one compatible entity? Do comprehenders then predict egocentrically and assume that the speaker is likely to refer to an entity that is compatible with their own perspective (in this case, their own gender)?

In fact, there is much evidence for egocentricity during language comprehension. In particular, research suggests that comprehenders consider objects as potential referents even though they know the speaker has no knowledge of these objects (an egocentric bias). For example, Keysar *et al.* [8–10] had a confederate instruct participants to reorganize objects in a grid. Participants knew that some objects (e.g. a small candle) were visible only to them, while others (medium and large candles) were visible to both them and the confederate. Even though participants knew that the confederate had no knowledge of the small candle, they often considered it as a potential referent when the confederate said *Now put the small candle above it*, and in fact reached for these objects nearly one-fourth of the time. These findings suggest that participants' egocentric biases (i.e. the intrusion of their own perception) affect language comprehension.

Thus, although Corps *et al*. [2] found that participants predicted consistently, in line with the agent's perspective, participants may predict egocentrically when the agent's perspective does not make it clear what to predict. We investigated this possibility using a procedure identical to Corps *et al*., but our sentences used the gender-neutral pronoun *They*. Research suggests that this pronoun is typically interpreted as gender-neutral (e.g. [11]), and so comprehenders will be unable to make gender-based predictions about what the speaker is likely to say. In particular, they will not know whether *They* is stereotypically masculine or stereotypically feminine.

Thus, male and female participants listened to male and female speakers producing sentences such as *They would like to wear…* while stereotypically masculine and feminine objects were displayed on-screen (two targets: a tie and a dress; two distractors: a drill and a hairdryer). The speakers arbitrarily referred to one of the two targets, and so participants could not learn to interpret *They* as having a particular gender in this experiment. We tracked participants' eye movements while they listened to these sentences to determine whether they predicted egocentrically. If they do, then we expect participants to fixate targets stereotypically compatible with their own gender (i.e. a tie when the comprehender is male; a dress when the comprehender is female) more than targets that are not compatible with their gender. These results would mirror those of Corps *et al*. [2], who found that participants predicted from their own perspective when the sentence explicitly referred to their perspective (e.g. *You would like to wear…*). Importantly, those findings suggest participants predicted consistently—participants were the agent of the sentence and predicted from the agent's perspective. If we find the same pattern in this experiment, these predictions would be egocentric because the participant is not the agent of the sentence.

But egocentric prediction may be inefficient. There is no reason why a speaker should produce an utterance that corresponds to what the comprehender would say, unless of course it happens to correspond to what the speaker would also say. If the speaker could plausibly refer to either target (i.e. by producing a sentence about an agent with the pronoun *They*), then egocentric prediction will be no more accurate than simply predicting that the speaker could refer to either target. Thus, participants may simply fixate the two targets equally, and more than the two distractors. Note that such predictions could be either associative (i.e. hearing *wear* and predicting wearable objects) or consistent (i.e. participants realize that *They* is gender-neutral and therefore predict that either target could occur), but importantly they cannot be egocentric. Alternatively, participants may simply fixate objects stereotypically compatible with the speaker's gender, which is highly salient from the speaker's voice (e.g. [7]). If this is the case, then we would expect the results to mirror Corps *et al*. [2]: participants would fixate targets stereotypically compatible with the speaker's gender (i.e. a tie when the speaker is male; a dress when the speaker is female) more than those that are incompatible.

Our discussion of gender refers to (cisgender) males and females and does not consider other gender identities (e.g. [12]), primarily because our participants identified themselves as either male or female and reported that their gender matched their birth gender. We also assume that our participants have gender-binary stereotypes. For example, a participant might consider a dress as stereotypically feminine; they could also consider it as stereotypically gender-neutral, but importantly they could not consider it as stereotypically of another gender (see [2]).

## 2. Method

### 2.1. Participants

We recruited 32 native English speakers (aged between 18 and 25; 16 males, 16 females) from the University of Edinburgh, who participated in exchange for £5. Participants did not have any known speaking, reading, or hearing impairments. All participants indicated their gender and whether they identified as the gender they were assigned at birth. These questions were open-ended (i.e. gender was not assumed to be binary), and so participants could answer in any way they wish. Importantly, all participants reported being male or female and identified as the gender that they were assigned at birth. Our sample size was based on previous studies using the visual-world paradigm with a similar design (in particular, [2,3]; see also [4]). The experiment was approved by the ethics committee at the University of Edinburgh.

### 2.2. Materials

We used the same stimuli as Corps *et al*. [2] (Experiment 1; a full list of stimuli can be found in appendix A), but we replaced the pronoun *I* with the pronoun *They*. In particular, participants heard 56 sentences, each paired with a display of four coloured objects. The sentences contained predictive verbs (e.g. *wear*), so that two of the four depicted objects were potential targets of the verb (e.g. a tie and a dress), while the other two were distractors (e.g. a drill and a hairdryer). Thus, participants heard sentences such as *They would like to wear…* . Corps *et al*. confirmed the predictability of these verbs in a separate pre-test, in which participants were presented with the sentences truncated at the final word. Participants selected which of four coloured objects they thought a speaker producing the sentence could refer to. Participants selected the two objects that were targets of the verb (i.e. the tie and the dress after reading *I would like to wear the nice…*) 96.5% of the time.

Twenty-eight of these sentences were gendered, so that two of the four objects were stereotypically feminine (e.g. one target: dress; one distractor: hairdryer), while the other two were stereotypically masculine (e.g. one target: tie; one distractor: drill). These assessments were based on extensive pre-testing (see [2, p. 5 and table 2]). Importantly, stereotypically masculine objects were considered just as masculine as stereotypically feminine objects were considered feminine.

The other 28 sentences were gender-neutral and were designed to make our gender manipulation less obvious to participants. These gender-neutral sentences contained predictive verbs (e.g. *eat*), but the four accompanying objects were rated as gender-neutral in Corps *et al*. [2]. Two of the objects were potential targets of the verb (e.g. apple and banana), while the other two were distractors (e.g. water and milk). We analysed these sentences to further test associative prediction.

Sentences were recorded by a native British English male speaker and a native British English female speaker. The male speaker was considered masculine and the female speaker was considered feminine by a separate group of participants in Corps *et al*. [2]. Importantly, the male speaker was considered just as masculine as the female speaker was considered feminine. The speakers produced the sentences at a natural rate. The pronoun *They* was designed to be gender-neutral, and we did not want participants to learn to associate *They* with a particular gender. As a result, there were four versions of each gendered sentence: (1) a male speaker referring to a stereotypically masculine target; (2) a male speaker referring to a stereotypically feminine target; (3) a female speaker referring to a stereotypically feminine target; and (4) a female speaker referring to a stereotypically masculine target. For the gender-neutral sentences, the speaker arbitrarily referred to one of the two targets, in a manner consistent across the two speakers (e.g. if the male speaker referred to *apple*, the female speaker also referred to *apple*). Sentences were between 2615 and 4382 ms. Sentences produced by the two speakers were matched for their duration, the onset and offset of the critical word, and the onset of the target (all *p* values > 0.13 in *t*-tests; table 1).

**Table 1.** The means (and standard deviations) of sentence duration, verb onset and offset, and target onset for the sentences produced by male and female speakers.

speaker gender	duration	verb onset	verb offset	target onset
male	3405 (346)	1621 (414)	1981 (386)	2742 (376)
female	3352 (339)	1532 (357)	1907 (345)	2696 (350)

### 2.3. Design

Speaker gender was manipulated within items and participants. Participants were randomly assigned to one of four stimulus lists, so that they heard only one version of each item, but heard 28 gendered sentences and 28 gender-neutral sentences. For the gendered sentences, seven had a male speaker refer to a stereotypically masculine object, seven had a male speaker refer to a stereotypically feminine object, seven had a female speaker refer to a stereotypically feminine object, and seven had a female speaker refer to a stereotypically masculine object.

Following Corps *et al*. [2], each object was shown twice: once as a target and once as a distractor. For the gendered sentences, each visual layout consisted of a stereotypically feminine target, a stereotypically masculine target, a stereotypically feminine distractor, and a stereotypically masculine distractor. For the gender-neutral sentences, participants were shown two targets and two distractors. Twenty-four layout combinations were used once, and four randomly selected layouts were used twice.

### 2.4. Procedure

We followed the same procedure as Corps *et al*. [2]. Participants were seated in front of a 1024 × 768 pixel monitor and were instructed to listen to the sentences while looking at the accompanying pictures. Eye movements were recorded using an Eyelink 2000 Tower mount eye-tracker sampling at 1000 Hz from the right eye and the experiment was run using ExperimentBuilder (SR Research). After reading the study instructions, participants placed their head on the chin rest and the eye-tracker was calibrated using a nine-point calibration grid.

**Figure 1.**
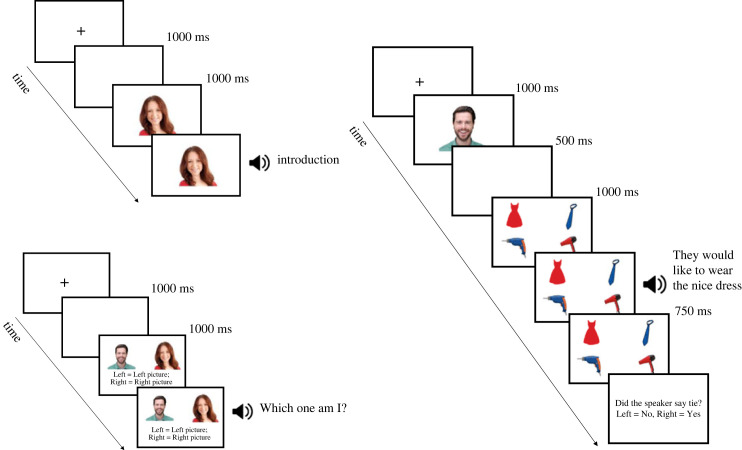
Schematic representation of the procedure for each phase of the experiment.

After calibration, participants were familiarized with the two speakers (figure 1). Each speaker introduced themselves once (with order counterbalanced across participants). Participants saw a fixation cross, which was followed by a blank screen displayed for 1000 ms. We then displayed the speaker's picture in the centre of the screen (at a size of 300 × 300 pixels) and the speaker then introduced themselves 1000 ms later by saying ‘Hi, I am Sarah/Andrew and you are going to hear me describe some objects. Please listen carefully and look at the objects on-screen’ (the top-left panel of figure 1). Participants were then asked to match each speaker's voice to their picture. Participants saw a fixation dot, followed by a blank screen displayed for 1000 ms. Both speakers' pictures were displayed in the centre of the screen (one on the left and one on the right, counterbalanced across participants), and each speaker asked ‘Which one am I?’ (with order counterbalanced; the bottom-left panel of figure 1). Participants then pressed a button on the button-box (left button for the speaker displayed on the left; right button for the speaker displayed on the right) to indicate which picture corresponded to the heard speaker. Participants always correctly identified the speaker from their voice.

In the main experiment (see the right panel of figure 1), each trial started with a fixation dot. A 300 × 300 pixel picture of the speaker was then displayed in the centre of the screen for 1000 ms. A blank screen was then displayed for 500 ms and the four pictures were presented in each of the four corners of the screen. Sentence playback began 1000 ms later, and the pictures remained on-screen for 750 ms after sentence end. After the sentence end, participants answered a comprehension question, which asked if the speaker referred to a particular object (e.g. *Did the speaker say hairdryer*?). The comprehension question mentioned an object the speaker referred to on half of the trials; for the other half of the trials, the question referred to one of the three unmentioned objects. Participants pressed the left button on the response box to answer yes and the right to answer no, and the next trial began immediately (without feedback). Participants completed four practice trials and were given the opportunity to take a break after 28 experimental trials.

### 2.5. Data analysis

We analysed the eye-tracking data in RStudio (version 2022.12.0 + 353) using the same procedure as Corps *et al*. [2]. Fixations to the four pictures were coded binomially (fixated = 1; not fixated = 0) for each 50 ms time bin from 1000 ms before to 1500 ms after verb onset. Participants fixated a picture if their fixations fell in the interest area of 300 × 300 pixels around the pictures. We coded blinks and fixations outside the interest areas as 0 (i.e. no fixation to any of the objects) and included them in the data. Our analysis focused on the gendered trials to determine whether participants predicted egocentrically, by fixating the target stereotypically compatible with their own gender over the target stereotypically compatible with the speaker's gender.

We analysed our data using bootstrapping analysis, which deals with the non-independence of fixations common in eye-tracking data. We chose bootstrapping over typical binning analysis because: (1) binning involves fitting as many models as there are time points, which increases the likelihood of a Type 1 error [13], and (2) fixations in adjacent bins are highly correlated. Furthermore, bootstrapping has worked well in our previous studies, which have used a similar approach (e.g. [2,3]). Bootstrapping identifies the time point at which looks to one object (or a set of objects) diverge from looks to another [14]. This analysis involves three steps. First, we apply a one-sample *t*-test to fixation proportions at each time point, aggregating over items. Average fixation proportions are compared to 0.50, with a significant *p*-value indicating that the object(s) attracted more than half of the fixations. A divergence point is then identified by determining the first significant time point in a run of at least ten consecutive significant time points (i.e. 500 ms). New datasets are then generated 2000 times, using a non-parametric bootstrap, which resamples data from the original dataset using participant, time point, and image type as categories. A new divergence point is estimated after each resample, and a mean is then calculated. Confidence intervals (CIs) indicate variability around the average divergence point. We ran the bootstrap from verb onset (0 ms) to 1500 ms after verb onset.

To determine whether participants predicted egocentrically, we compared fixations to the target compatible with the participant's gender (e.g. the tie if the participant was male) to the target incompatible with the participant's gender (the dress). This analysis was based on all the gendered trials, which were those in which the participant and the speaker had the same gender (the gender-match trials) and the ones in which they had different genders (the gender-mismatch trials). To preview our results, we found no evidence that participants were egocentric in their predictions. However, it is possible that any egocentricity was drowned out by predictions made based on the speaker's voice, which was a highly salient cue and available before verb onset (e.g. [7]). We tested this possibility in a second analysis, in which we compared looks to the participant-compatible target to looks to the participant-incompatible target for the gender-mismatch trials only. Running a comparable analysis on the gender-match trials was not necessary for testing our predictions, since these trials do not isolate egocentric prediction, but we conducted an identical analysis on the gender-match trials for the sake of completeness.

Both of these analyses tested whether participants predicted egocentrically. But we also tested whether they predicted that the agent could prefer either of the two targets over the two distractors (e.g. looking at wearable objects after hearing the verb *wear*). These predictions could be either associative (i.e. hearing *wear* and predicting wearable objects) or consistent (i.e. participants realize that *They* is gender-neutral and therefore predict that either target could occur), but importantly prediction in these instances cannot be egocentric. To determine whether such predictions occurred, we compared fixations to the two targets to fixations to the two distractors. In particular, fixations were coded as 1 if participants fixated either of the targets, and 0 if they did not. We conducted this analysis for both the gendered and gender-neutral trials separately. Raw data and scripts for all analyses are available on Open Science Framework at: https://osf.io/sq2wm/.

## 3. Results

### 3.1. Comprehension question accuracy

Participants correctly answered the comprehension questions on 99% of the trials, suggesting they paid attention to the sentences and pictures.

### 3.2. Eye-tracking data

**Figure 2.**
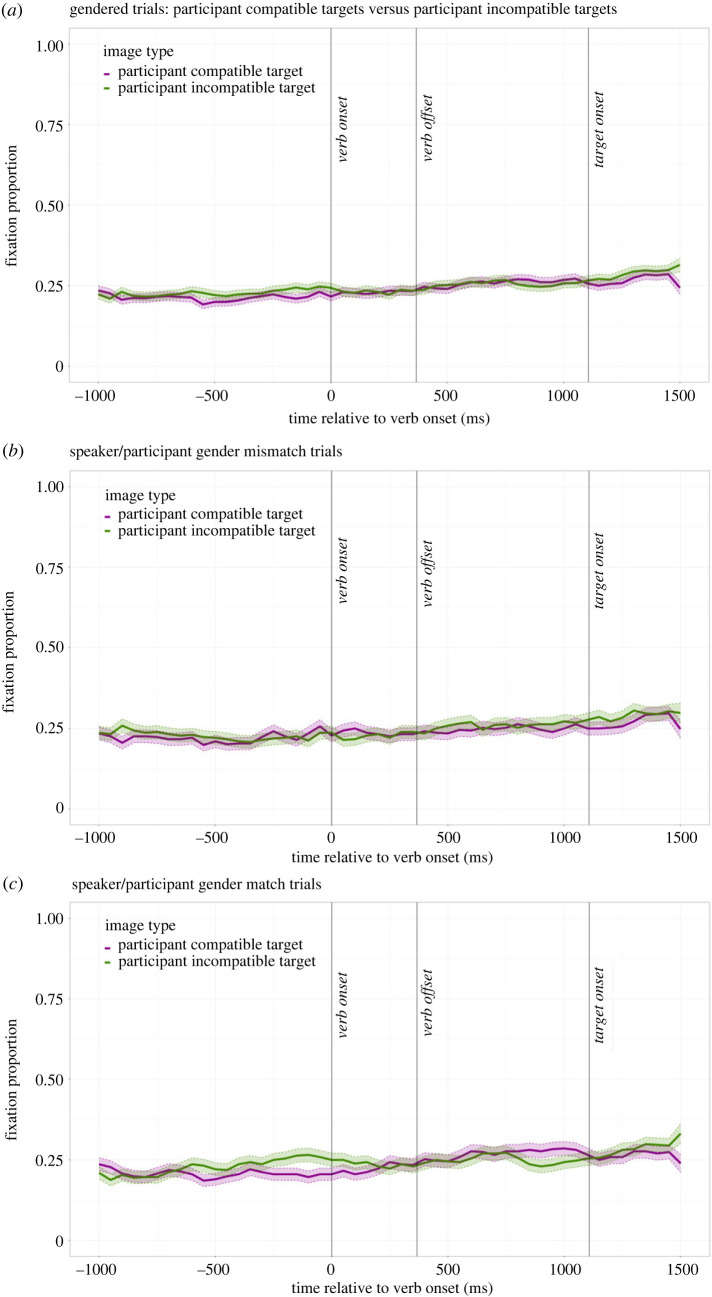
Eye-tracking results for the gendered trials. (*a*) The average fixation proportions to the participant-compatible and participant-incompatible targets for all gendered trials. (*b*,*c*) The average fixation proportions to these targets for the gender-mismatch trials (speaker and participant had different genders; *b*) and the gender-match trials (speaker and participant had the same genders; *c*). Transparent thick lines are error bars representing standard errors.

Note that we analysed our data using the same procedure as Corps *et al*. [2], and so there is overlap in how we report our results. Figure 2 shows the average fixation proportions on the participant-compatible and participant-incompatible targets for all the gendered trials (figure 2*a*) and for the gender-mismatch (participant and speaker had different genders; figure 2*b*) and gender-match (participant and speaker had the same genders; figue 2*c*) trials separately. In the bootstrapping analysis, we found no evidence that participants ever predicted egocentrically—there was no timepoint at which the participant-compatible target attracted more fixations than the participant-incompatible target (figure 2*a*). This result was further confirmed when we analysed the gender-mismatch (figure 2*b*) and gender-match (figure 2*c*) trials separately, with the gender-mismatch trials suggesting egocentric effects were not drowned out by any predictions participants may have made simply using the speaker's voice. Thus, we found no evidence that participants predicted egocentrically, from their own perspective.

**Figure 3.**
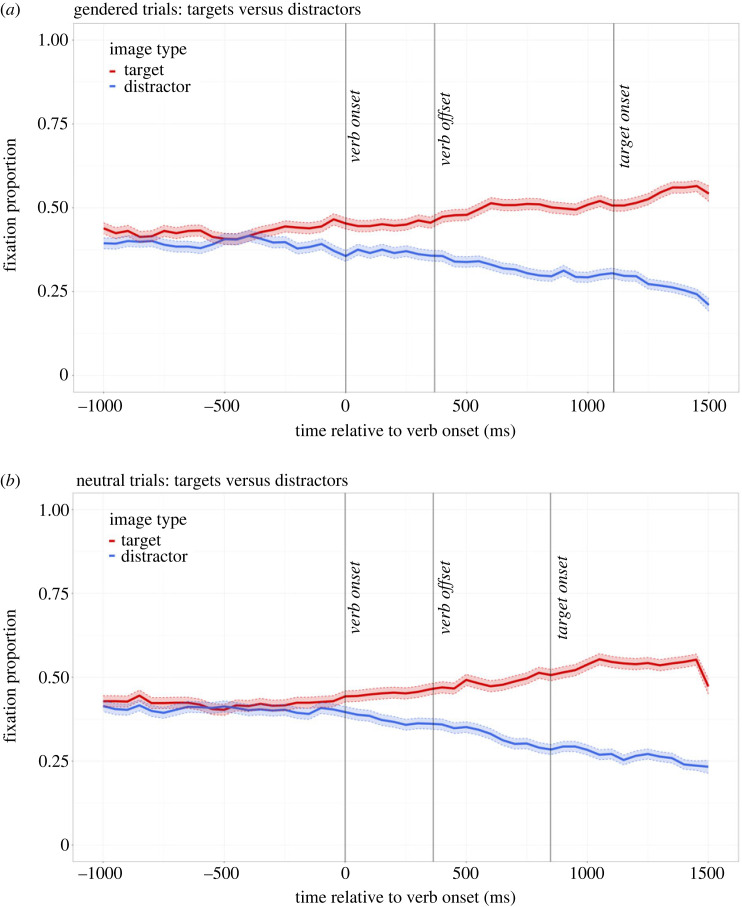
Eye-tracking results for the gendered (*a*) and neutral (*b*) trials. (*a*) The average fixation proportions on the two targets and the two distractors for all gendered trials. (*b*) The same results for the neutral trials. Transparent thick lines are error bars representing standard errors.

Figure 3 shows the average fixation proportions on the two targets and the two distractors for the gendered (figure 3*a*) and neutral (figure 3*b*) sentences. The bootstrapping analysis showed that participants fixated the two targets more than the two distractors from 467 ms (CI [350, 700]) after verb onset for the gendered trials (figure 3*a*) and 515 ms (CI [400, 700]) after for the neutral trials (figure 3*b*). The CI does not contain zero, and so supports a reliable difference between the two groups of objects. Thus, we found no evidence that participants predicted egocentrically, but they did predict that the agent would prefer targets over distractors. Importantly, the time-courses of these predictions are in line with our previous studies where we have compared looks to targets to looks to distractors. For example, we found that L1 participants fixated targets more than distractors from 519 ms after verb onset [2; Experiment 1], and L2 participants fixated targets more than distractors from 527 ms after verb onset [3]^1^.

## 4. Discussion

In this study, we investigated whether comprehenders predict egocentrically when they do not have a strong sense of what the speaker is likely to refer to. In particular, we tested whether participants would be egocentric in their predictions when they listened to sentences containing the gender-neutral pronoun *They* (e.g. *They would like to wear…*) and there were two plausible targets. We found no evidence that participants ever predicted egocentrically (e.g. hearing *wear* and then fixating stereotypically masculine targets if the participant was male). However, participants predictively fixated objects semantically associated with the critical verb (i.e. fixating the two targets over the two distractors), thus indicating that they do make predictions under such circumstances. These predictions could be associative (i.e. hearing *wear* and predicting wearable objects) or consistent (i.e. participants realize that *They* is gender-neutral and therefore predict that either target could occur), but importantly they cannot be egocentric.

In our previous experiment [2; Experiment 1], associative prediction occurred within 519 ms after verb onset, while consistent prediction, based on the agent's perspective, occurred later (within 641 ms after verb onset). In the current study, participants fixated the two targets more than the two distractors from 467 ms after verb onset in the gendered trials and 515 ms for the gender-neutral trials. Thus, the time-courses of these predictions are consistent with the time-course of associative prediction, supporting the suggestion that participants heard the verb *wear* and predicted that the speaker would refer to wearable objects. Note, however, that the analysis in this experiment was based on twice the number of observations as in Experiment 1 in Corps *et al*., which may have affected the time-course of prediction, presumably making the difference between conditions emerge somewhat earlier. Nevertheless, our findings do not support the suggestion that participants heard the pronoun *They*, realized that this pronoun was gender-neutral, and then predicted that the agent could prefer either object. This pattern would reflect consistent prediction, which we would expect to occur much later than associative prediction. If participants had predicted in this way, then we would expect a difference in the time-course of prediction in the gendered and the gender-neutral trials: none of the objects were gendered in the gender-neutral trials, and so participants could rely only on associative predictions.

Our findings are strikingly different from research demonstrating that participants initially comprehend egocentrically, before incorporating contextual information (such as another person's perspective; e.g. [15]). However, these studies differ from our experiment in two critical ways. First, they focused on egocentric bias, and particularly whether one's own beliefs or perceptions (such as whether an object can be seen or not) affect processing when trying to be objective about another person's perspective. In our study, we tested whether participants incorporate another person's perspective into their predictions, thus focusing on egocentric prediction. Second, previous studies have also focused on bottom-up comprehension, while we focused on top-down prediction. The findings therefore suggest that top-down and bottom-up processing draw on different mechanisms. Consistent with this suggestion, Barr [16] found that participants were four times more likely to fixate objects in common ground (visible to both the participant and their partner) than objects in privileged ground (visible only to the participant) before they started processing the object name, suggesting they predicted from their partner's perspective. But Barr's participants also showed phonological interference effects from the competitor, regardless of whether it was in common or privileged ground, suggesting that perspective did not constrain bottom-up lexical processing.

Participants could have also predicted associatively simply using the speaker's voice. For example, Borovsky & Creel [7] familiarized participants with two talkers, such as a pirate and a princess, whose roles were strongly associated with particular objects. Participants predictively fixated objects compatible with the speaker's role; for example, if they heard the pirate speaking then they fixated the sword and the ship more than the wand and the carriage. These fixations occurred from the very start of the trial, even before participants heard a predictive verb (e.g. *hold*), suggesting that participants expected the speaker to refer to objects that had been explicitly associated with their voice during training. We found no evidence for such associative prediction in our study: participants did not fixate objects just because they were stereotypically compatible with the speaker's voice (see figure 2).

It is worth noting that finding gender-based egocentric prediction rests on the assumption that male participants will prefer stereotypically masculine objects, while females will prefer stereotypically feminine objects. We did not assess participants' individual preferences, but our previous studies suggest this assumption is reasonable. In particular, in Corps *et al*. [2], we found that participants tended to fixate objects stereotypically compatible with their own gender when the participant was the agent of the sentence and it explicitly highlighted their perspective (e.g. *You would like to wear…*). Thus, the lack of egocentric prediction in this study cannot be attributed to the fact that the participants may not have preferred objects stereotypically compatible with their own gender.

Participants could also interpret *They* as a gender-neutral, but plural pronoun. This interpretation would be incompatible with many of the sentences (e.g. *They would like to wear the nice dress*) or pictures (e.g. a single dress). But even if participants did sometimes interpret *They* as plural, the experiment would still demonstrate that participants did not predict egocentrically.

In sum, we used the visual-world eye-tracking paradigm to investigate whether comprehenders predict egocentrically when the agent's perspective (i.e. their gender identity) does not make it clear what to predict. Participants heard sentences about a gender-neutral entity (e.g. *They would like to wear…*) while viewing four objects on-screen (a tie, a dress, a drill, and a hairdryer). We found no evidence that participants ever predicted egocentrically, from their own perspective. However, participants did predict that the agent would prefer the two targets over the two distractors. These predictions could be associative (i.e. hearing *wear* and predicting *wearable* objects) or consistent (i.e. understanding that *They* is gender-neutral and therefore predicting either target could occur), but importantly they are not egocentric. These findings suggest that participants do not fall back on their own egocentric perspective when predicting, even when the agent's perspective does not make it clear what to predict.

## Data Availability

Raw data and analysis code can be accessed at: https://osf.io/sq2wm/.

## Footnotes

1 Note that effects are likely slightly earlier in this study because this analysis was on twice the data. In our previous studies, we compared one target to one distractor, but here we compared both targets to both distractors.

## Appendix A

Gendered and gender-neutral sentence fragments and target picture names used in the experiment. Predictive verbs are highlighted in bold.

See tables 2 and 3.

**Table 2.** Gendered sentences used in the eye-tracking experiment. The speaker referred to either target.

sentence	masculine target	feminine target	masculine distractor	feminine distractor
They went to dinner last night and **wore** a nice	shirt	corset	builder	mermaid
They decided not to **wear** the nice	turban	makeup	truck	doll
They really wanted to **become** a good	king	princess	tie	dress
They would really like to **buy** the nice	barbeque	roses	mechanic	cheerleader
They have decided to **buy** a nice	wallet	necklace	firefighter	ballerina
They have decided to **wear** the new	belt	perfume	chainsaw	tweezers
They once dreamed about **becoming** a nice	knight	nun	waistcoat	cardigan
Today, they will **wear** the new	vest	skirt	hammer	hairbrush
Later on, they will **use** a great	drill	hairdryer	beer	cocktail
Tonight, they will **wear** the nice	cufflinks	earrings	digger	pram
Later on today, they will **purchase** a nice	kilt	ring	pirate	witch
Later, they will go out and **buy** the great	gun	diamond	plumber	nurse
Tonight, it is likely they will **wear** a great	tie	dress	drill	hairdryer
They would really like to **drink** the nice	beer	cocktail	turban	makeup
Later, they are going to **use** the new	urinal	tampon	king	princess
In the evening, they will **play** some good	golf	volleyball	cufflinks	earrings
They used to dream about **becoming** a great	pirate	witch	wallet	necklace
They had a dream about **becoming** a great	builder	mermaid	vest	skirt
When they go out, they will **carry** a nice	briefcase	handbag	shirt	corset
They have decided to **become** a good	mechanic	cheerleader	kilt	ring
They used to dream of **becoming** a great	plumber	nurse	briefcase	handbag
They would not like to **wear** the nice	tuxedo	earmuffs	barbeque	roses
They will go out and **buy** the nice	hammer	hairbrush	knight	nun
When they were younger, they liked to **push** the new	digger	pram	urinal	tampon
They used to enjoy **playing with** the nice	truck	doll	belt	perfume
They will go out and **help** the nice	firefighter	ballerina	tuxedo	earmuffs
Today, they would like to **wear** the nice	waistcoat	cardigan	gun	diamond
They have decided to **use** the nice	chainsaw	tweezers	golf	volleyball

**Table 3.** Gender-neutral sentences used in the eye-tracking experiment. The speaker referred to one of the two targets, but this target was the same for a male and female speaker.

sentence	target 1	target 2	distractor 1	distractor 2
Later on, they will **eat** the nice	apple	banana	water	milk
They are going to **eat** the nice	cookie	donut	hoodie	socks
They have decided that they will **wear** the great	trainers	wellies	cake	mushroom
They have decided to **eat** the nice	kiwi	carrot	hat	glasses
Later, it is likely that they will **eat** the nice	bread	pie	bed	toaster
They once thought about **becoming** a good	dentist	optician	toothbrush	pencil
They would like to **become** a great	chef	vet	coffee	tea
They have decided to **eat** some nice	chocolate	spaghetti	tennis	badminton
They would like to **eat** some good	popcorn	cereal	headphones	gloves
They are going to **feed** the nice	parrot	zebra	poncho	dungarees
They would like to **eat** a great	pumpkin	tomato	jumper	suitcase
They thought about **becoming** a great	doctor	photographer	computer	piano
Tomorrow, they will **visit** the nice	pyramids	volcano	bread	pie
They would like to **wear** the nice	headphones	gloves	cookie	donut
Today, they will **wear** the new	hat	glasses	kiwi	carrot
They would like to **drink** some great	water	milk	chocolate	spaghetti
This afternoon, they will **drink** a great	coffee	tea	monkey	tiger
They will go out later and **wear** the nice	hoodie	socks	pumpkin	tomato
They would like to **play** some great	tennis	badminton	popcorn	cereal
Later today, they will go out and **buy** a new	bed	toaster	chef	vet
They need to go out and **buy** a new	jumper	suitcase	dentist	optician
Later, they will **buy** a new	computer	piano	doctor	photographer
Tomorrow, they will **wear** the new	poncho	dungarees	pancakes	cheese
Tomorrow, it is likely that they will **eat** a nice	cake	mushroom	parrot	zebra
They have decided that I will **feed** the nice	monkey	tiger	earplugs	medal
They would like to **use** the nice	toothbrush	pencil	pyramids	volcano
They have decided to **wear** the nice	medal	earplugs	apple	banana
Later, they will **eat** the new	pancakes	cheese	trainers	wellies
